# Embedded Implementation of VHR Satellite Image Segmentation

**DOI:** 10.3390/s16060771

**Published:** 2016-05-27

**Authors:** Chao Li, Souleymane Balla-Arabé, Dominique Ginhac, Fan Yang

**Affiliations:** Laboratory LE2I UMR 6306, CNRS, Arts et Métiers, University Bourgogne Franche-Comté, 21000 Dijon, France; balla_arabe_souleymane@ieee.org (S.B.-A.); dom@le2i.cnrs.fr (D.G.); fanyang@u-bourgogne.fr (F.Y.)

**Keywords:** high-level synthesis, SW/HW co-design, remote sensing, high performance computing, embedded system, FPGA, image segmentation, level set method, active contour model, lattice Boltzmann method

## Abstract

Processing and analysis of Very High Resolution (VHR) satellite images provide a mass of crucial information, which can be used for urban planning, security issues or environmental monitoring. However, they are computationally expensive and, thus, time consuming, while some of the applications, such as natural disaster monitoring and prevention, require high efficiency performance. Fortunately, parallel computing techniques and embedded systems have made great progress in recent years, and a series of massively parallel image processing devices, such as digital signal processors or Field Programmable Gate Arrays (FPGAs), have been made available to engineers at a very convenient price and demonstrate significant advantages in terms of running-cost, embeddability, power consumption flexibility, *etc*. In this work, we designed a texture region segmentation method for very high resolution satellite images by using the level set algorithm and the multi-kernel theory in a high-abstraction C environment and realize its register-transfer level implementation with the help of a new proposed high-level synthesis-based design flow. The evaluation experiments demonstrate that the proposed design can produce high quality image segmentation with a significant running-cost advantage.

## 1. Introduction

Remote sensing techniques are increasingly used in geological exploration, natural disaster prevention, monitoring, *etc*. They usually require very high resolution satellite images, which are texturally-rich and may raise the running-cost of systems. In some cases, for example during volcano eruptions or flooding monitoring, fast and effective processing methods are necessary because the information needs to be extracted and considered as fast as possible. However, image segmentation models usually used to detect objects or other relevant features in very high resolution satellite images are fundamentally time consuming, which is a real hassle for researchers.

Meanwhile, parallel computing techniques and embedded systems have made great progress in recent years. A series of highly parallelized image processing devices have been made available to engineers at a very affordable price. Such devices have been widely used in various signal processing and communication systems for their significant advantages in terms of running-cost, embeddability, power consumption or flexibility [1,2,3,4,5,6,7]. For example, Zeng Yonghong [7] presented an efficient Intellectual Property (IP) core design methodology to implement a real-time image processing application, such as the Normalized Product correlation (NProd) image matching algorithm; Chiesi *et al.* [2] proposed a new non-conventional technique based on Fuzzy Deconvolution for Scattering Center Detection (F-SCD) and its embedded implementation for real-time deployment in an automotive collision avoidance application. Komuro *et al.* [8] designed an architecture of embedded systems for high-frame-rate real-time vision on the order of 1000 f/s, achieving both hardware reconfigurability and easy algorithm implementation, while fulfilling performance requirements. Moreover, many comparative studies indicate that Field Programmable Gate Arrays (FPGAs) can often achieve better comprehensive properties than other platforms in most cases. For example, in the work of Zou *et al.* [9], the efficiency of the FPGA implementation of the Smith–Waterman Algorithm is 3.4× compared to the Graphics Processing Unit (GPU) and over 40× compared to the Central Processing Unit (CPU), while Kestur *et al.* [10] demonstrated that FPGA has similar performance at higher energy efficiency compared to the CPU and GPU platforms.

In this work, we attempt to design and implement a high performance image segmentation application for satellite image processing systems using FPGAs. First of all, we base the design on a promising level set method segmentation dedicated to very high resolution satellite images [11,12,13]. Next, the Lattice Boltzmann Method (LBM) is used as the solver of the level set equation for its highly parallelizable capability. Finally, we successfully implement the algorithm into the Register-Transfer Level (RTL) for FPGAs. The contributions of this work include:

(1) a highly parallel image segmentation algorithm dedicated to very high resolution satellite images [14,15] is prototyped and validated,
(2) the implementation process of this algorithm into the register-transfer level is described,
(3) a high-level design flow is developed by using the recent high-level synthesis tools to improve the development productivity and maintainability of the design,
(4) a series of optimizations are sequentially made in the routine level to reduce the running-cost of the design; the experiments demonstrate that a significant performance improvement is achieved.

In the experiments, we first evaluate the performance of the proposed implementation and the optimizations applied during the synthesis process. Next, two reference implementations are made by using MATLAB and C for a comparison evaluation. Finally, the development cycles are studied for development productivity analysis. The results demonstrate that the applied design flow is an effective method to facilitate the implementation on FPGA. The optimizations made during the synthesis significantly accelerate the designs by parallelizing the operations and simplifying the control flow of the generated register-transfer level implementation. According to the comparison experiments, the proposed implementation achieves a speedup of over 10× compared to the CPUs.

The remainder of this paper is organized as follows: Section 2 introduces briefly the background of this research; Section 3 describes the selected segmentation algorithm for very high resolution satellite images; Section 4 presents the development framework specially designed for the algorithm of this paper; Section 5 presents the prototype of the target algorithm and its optimized implementation; Section 6 analyzes the experiment procedure and the results; finally, a conclusion is given in Section 7.

## 2. Background

The level set method refers to the class of active contour models that uses the implicit representation of the evolving curve instead of the parametric one, *i.e*., the Lagrangian framework and Eulerian framework [16,17]. Its advantages include the following:

(1) It allows the texture of interest to be selected and segmented depending on the user requirements by providing a set of user-controlled parameters,
(2) It allows easier handling of complex shapes and topological changes,
(3) It can convert the images from 2D to 3D straightforwardly,
(4) It allows constraints on the smoothness of the boundaries to be added easily via some regularization terms.

The basic idea of the level set method is to continuously evolve the zero level of a given level set function in the image domain until it reaches the boundaries of the interested regions or objects. First of all, we define a closed curve in the image. Next, the distance between the boundary of this curve and each pixel, d(x,y), is expressed by using a signed distance function:

(1) d(x,y)=ϕ(x,y,t)

where (x,y) refers to the pixel and *t* is time. We can see that at any time *t*, the pixels on the curve always satisfy:

(2) ϕ(x,y,t)=0

Differentiating the equation above with respect to *t*, we can get:

(3) $$\phi_{t}+\phi_{x}\frac{dx}{dt}+\phi_{y}\frac{dy}{dt}=0$$

According to the chain rule, we can figure out the following equation:

(4) $$\frac{\partial \phi }{\partial t}=V\left|\nabla \phi \right|$$

where ϕ,∇ϕ and *V* are respectively the level set function, the gradient of ϕ and the speed function that should drive and attract the evolution of the active contour toward the object boundaries. Equation (4) is known as the level set equation, which can be used to govern the active curve evolution. According to whether the speed function uses local or regional statistics, the level set methods can be divided into two general approaches: edge-based and region-based methods. The first one uses an edge indicator depending on the gradient of the image as in the classical snakes, but these methods are effective only when the boundaries of the object have a clear change in gray value. Moreover, they are sensitive to noise and ineffective when the object of interest is without edges. The second one uses some regional attributes to stop the evolving curve. These methods are robust against noise and effective even if the object is without edge.

Up to present, many efforts have been made to apply the geometric active contour frameworks to the field of remote sensing. For example, Karantzalos *et al.* [18] propose a variational geometric level set functional for man-made object detection from aerial and satellite images, while Ball *et al.* [19] present a supervised hyper-spectral classification procedure consisting of an initial distance-based segmentation method that uses best band analysis, followed by a level set enhancement that forces localized region homogeneity. However, all of these methods are not local and, therefore, cannot benefit from the high performance computing techniques. The main limitation is due to the fact that, at each iteration, the average intensities inside and outside the contour have to be calculated, increasing thus dramatically the processing time by increasing communications between processors.

Recently, some researchers pointed out that the lattice Boltzmann method is a potential approach to solve the level set equations [20]. This method was originally designed to simulate Navier–Stokes equations for an incompressible fluid [15,21,22] and used in image segmentation only recently [23]. Differing from the other solvers [18,19], the lattice Boltzmann method exhibits local and not global computations, which may result in less data dependencies than the other methods. Given the processing devices available today, such as FPGAs and GPUs, that features powerful parallel programming, this method appears as a natural candidate to improve the segmentation performance because of numerous advantages, such as simplicity, intrinsic massively parallel nature and second-order accuracy both in time and space.

In the design of this paper, FPGA is selected as the target device for its advantages in running-cost, embeddability, power consumption and flexibility. It is a reconfigurable device containing an array of programmable logic blocks and exhibiting a quite different design philosophy than the general-purposed processors based on the von Neumann or Harvard structures. Therefore, it does not run a program written by software designers and stored in the program memory, but needs to be configured in the register-transfer level using a hardware description language that allows one to create complex combinational functions by connecting the logic blocks. FPGAs are traditionally used for complex applications requiring a performance profile that cannot be easily achieved by any other processing device. In our case, the desired algorithm is architecturally complex, computationally intensive and massively parallelizable. Consequently, it is a nice candidate for an FPGA implementation, but its implementation and optimization in register-transfer level are far from easy. Therefore, to fix this issue, we base our design on the High-Level Synthesis (HLS) SW/HW co-design framework. The key point of this approach is to incorporate the high-level synthesis process into the design flow to automatically generate register-level languages from C-like languages, which have a higher abstraction level related to the former [24]. It is highly promising for complex FPGA designs [25,26,27,28]. It can effectively raise the development productivity by enabling the users to focus only on the algorithm level instead of the hardware level constraints, such as data flow, logic control or interface protocol. Furthermore, its synthesis process offers nice opportunities to parallelize the algorithms at different granularities (function, loop and instruction level), which may lead to an efficient implementation of the lattice Boltzmann method in our case.

## 3. Level Set Algorithm Description

### 3.1. Level Set Equation

In this work, we perform the following speed function by combining the level set method with multi-kernel theory:

(5) $$V\left(x\right)=\lambda \left(\varepsilon -{\left(T\left(\psi \left(x\right)\right)-T\left(I_{t}\right)\right)}^{2}\right)+V_{reg}\left(x\right)$$

with:

(6) $$\psi \left(x\right)=\left(I\left(x\right),I_{f}\left(x\right),s\left(x\right)\right)\in R^{3}$$

where *x* and $I_{t}$ are respectively the spatial variable and the intensity of a given pixel in a region of interest, *λ* is a user-controlled positive parameter used to accelerate the convergence of the proposed method toward the steady state, *ε* is the threshold parameter to stop the evolution, $V_{reg}$ is a regularization term, I(x)∈R is the intensity of the pixel *x*, the two-tuple $\left(I_{f}\left(x\right),s\left(x\right)\right)\in R^{2}$ is a simple descriptor of the texture information at the pixel *x*, in which $I_{f}\left(x\right)$ is the filtered intensity of the pixel *x* and s(x) is the standard variance of the intensities of the neighbors of *x*, and *T* is a transformation function defined as follows:

(7) K(x,y)=<T(x),T(y)>

where *K* is a nonnegative combination of two Mercer kernels $k_{1}$ and $k_{2}$:

(8) $$K=k_{1}\left(x,y\right)+\alpha k_{2}\left(x,y\right)\;\text{with}\;\alpha >0$$

The most commonly-used kernels include linear, polynomial and Gaussian kernels. In our case, we use the Gaussian kernel and define $k_{1}$ as the pixel intensity kernel, while $k_{2}$ is the texture information kernel. They are expressed as follows:

(9) $$\begin{array}{cc} & k_{1}\left(x,y\right)={exp\{-||I\left(x\right)-I\left(y\right)||}^{2}/\sigma^{2}\}\\ & k_{2}\left(x,y\right)=exp\{-||\left(I_{f}\left(x\right),s\left(x\right)\right)-\left(I_{f}\left(y\right),s\left(y\right)\right){\left|\right|}^{2}/\sigma^{2}\} \end{array}$$

where *σ* is the Gaussian root mean square width of the kernel.

On the other hand, the regularization term $V_{reg}\left(x\right)$ is defined as:

(10) $$V_{reg}\left(x\right)=\beta \left(1-\mu \left(x\right)\right)+\nu div\left(\nabla \phi /|\nabla \phi |\right)$$

where div is the divergence operator, *β* controls the impact of the texture information on the segmentation results, *ν* is a positive constant and *μ* is a fuzzy membership value that helps with deciding if the current pixel *x* is a boundary or not. *μ* is defined as follows:

(11) $$\mu \left(x\right)=\left\{\begin{array}{cc} 1-\frac{\eta -\delta }{\eta },\; & \eta ⩾\delta \\ 1, & \text{else} \end{array}\right.$$

with

(12) $$\begin{array}{c} \delta =|I\left(x\right)-\bar{I}\left(x\right)| \end{array}$$

where $\bar{I}$ is the mean intensity of the local pixels defined as:

(13) $$\bar{I}\left(x\right)=\frac{\int_{\Omega }s\left(x,y\right)I\left(y\right)dy}{\int_{\Omega }\left(x,y\right)dy}$$

with:

(14) $$s\left(x,y\right)=\left\{\begin{array}{cc} 1,\; & |x-y|<r\\ 0, & \text{else} \end{array}\right.$$

where *r* is the radius constant, *η* is a positive small parameter defined as 0.1 in this paper and Ω is the image domain. Consequently, the first term of $V_{reg}$ in Equation (10) drives the active contour as close as possible to those pixels with which μ(x)=1; and the second term is the constraint on its length.

Since the proposed speed function tends to zero when ${\left(T\left(\psi \left(x\right)\right)-T\left(I_{t}\right)\right)}^{2}$ tends to *ε* [11], which is a small value, the evolving contour will stop at a certain pixel whose intensity and texture information are equal to those of the selected region. Now, we can figure out the desired multi-kernel level set equation according to Equations (5)–(10):

(15) $$\begin{array}{cc} \frac{\partial \phi }{\partial t}= & \lambda \left(\varepsilon -2\left(1+\alpha -K\left(\psi \left(x\right),I_{t}\right)\right)\right)+\beta \left(1-\mu \left(x\right)\right)+\nu div\left(\nabla \phi \right) \end{array}$$

**Proof of Equation (15):**

(16) $$\begin{array}{c} According\;to\;Equation\;(5),\;we\;have:\\ V\left(x\right)=\lambda \left(\varepsilon -\left(K\left(\psi \left(x\right),\psi \left(x\right)\right)+K\left(I_{t},I_{t}\right)-2K\left(\psi \left(x\right),I_{t}\right)\right)\right)+V_{reg}\left(x\right)\\ ∵\text{Equations}\;(7)–(9)\Rightarrow \left\{\begin{array}{c} K\left(\psi \left(x\right),\psi \left(x\right)\right)=K\left(I_{t},I_{t}\right)=1+\alpha \\ K\left(\psi \left(x\right),I_{t}\right)=T\left(\psi \left(x\right)\right)\cdot T\left(I_{t}\right) \end{array}\right.\\ ∴V\left(x\right)=\lambda \left(\varepsilon -2\left(1+\alpha -K\left(\psi ,I_{t}\right)\right)\right)+V_{reg}\left(x\right)\\ ∵\text{Equation}\;(10),\;|\nabla \phi |=1,\;\text{and}\;V\left(x\right)=\frac{\partial \phi }{\partial t}\\ ∴\frac{\partial \phi }{\partial t}=\lambda \left(\varepsilon -2\left(1+\alpha -K\left(\psi \left(x\right),I_{t}\right)\right)\right)+\beta \left(1-\mu \left(x\right)\right)+\nu div\left(\nabla \phi \right) \end{array}$$

### 3.2. LBM Solver

**Figure 1:**
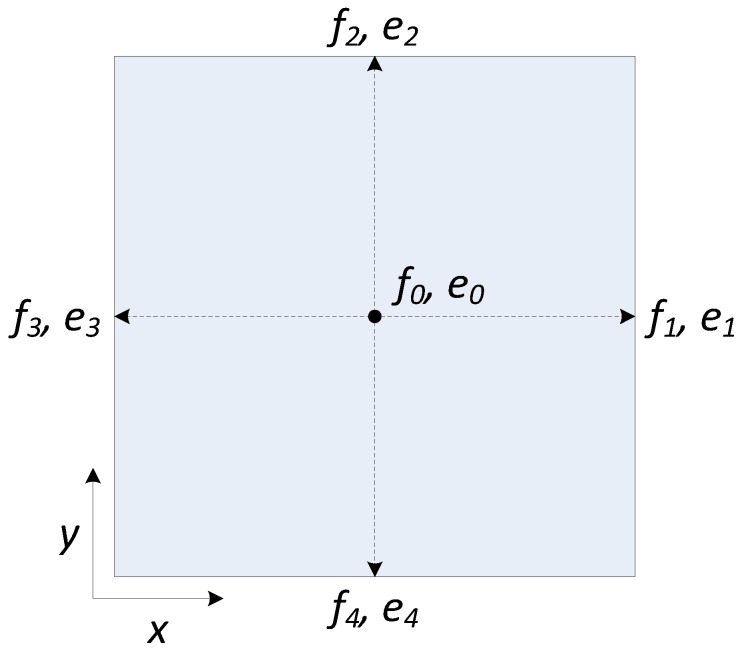
The D2Q5 Lattice Boltzmann Method (LBM) model.

In our case, the D2Q5 (two dimensions and five discrete velocity directions) LBM lattice structure shown in Figure 1 is used for the solver of the proposed level set equation. The level set equation evolution equation can be expressed as follows:

(17) $$f_{i}\left(r+e_{i},t+1\right)=f_{i}\left(r,t\right)+\Omega_{BGK}+\frac{D}{bc^{2}}\cdot F\cdot e_{i}$$

which can be considered as the following two steps:

(18) $$\begin{array}{cc} & \text{Collision}:\;f_{coll}\left(r,t\right)=f_{i}\left(r,t\right)+\Omega_{BGK}+\frac{D}{bc^{2}}\cdot F\cdot e_{i}\\ & \text{Streaming}:\;f_{i}\left(r+e_{i},t+1\right)=f_{coll}\left(r,t\right) \end{array}$$

where $e_{i}$ and $f_{i}$ are the velocity vector at each link of the model shown in Figure 1, the particle distribution that moves along the corresponding link, ***r*** is the position of the cell, *t* is time, ***F*** is an external force, D=2 is the grid dimension, b=5 is the link at each grid point, c=1 is the link length and $\Omega_{BGK}$ is the Bhatnagar–Gross–Krook collision model [29] expressed as:

(19) $$\Omega_{BGK}=\frac{1}{\tau }\left(\bar{f_{i}}\left(\vec{r},t\right)-f_{i}\left(r,t\right)\right)$$

where *τ* is the relaxation time and $\bar{f_{i}}$ is the local Maxwell–Boltzmann equilibrium particle distribution function. The continuous of $\bar{f_{i}}$ can be respectively expressed as:

(20) $$\bar{f_{i}}=\rho {\left(2\pi RT\right)}^{-3/2}exp\left\{-{\left(v-u\right)}^{2}/2RT\right\}$$

where ***v***, ***u*** and *ρ* are the particle velocity, macroscopic velocity and macroscopic fluid density. RT is the product of the gas constant and thermodynamic temperature. In the work of Y. Zhao [15], this equilibrium distribution can be expressed in discrete form as follows when modeling a typical diffusion phenomenon:

(21) $$\bar{f_{i}}\left(\rho \right)=\rho A_{i}\;\text{with}\;\rho =\sum_{i}f_{i}$$

where $A_{i}$ is a constant scalar coefficient specific to the chosen lattice geometry. According to the Chapman–Enskog analysis, the following diffusion equation can be obtained from the LBM evolution equation [15]:

(22) $$\frac{\partial \rho }{\partial t}=\xi div\left(\nabla \rho \right)+F$$

where *ξ* is the diffusion coefficient. It is obvious that Equation (22) is similar to Equation (15) with the body force expressed as follows:

(23) $$F=\lambda \left(\varepsilon -2\left(1+\alpha -K\left(\psi \left(x\right),I_{t}\right)\right)\right)+\beta \left(1-\mu \left(x\right)\right)$$

Now, we can obtain the desired level set equation solver by substituting $\Omega_{BGK}$ and $F\cdot e_{i}$ for Equations (19) and (23) in Equation (17):

(24) $$f_{i}\left(r+e_{i},t+1\right)=f_{i}\left(r,t\right)+\frac{1}{\tau }\left(\bar{f_{i}}\left(\vec{r},t\right)-f_{i}\left(r,t\right)\right)+\frac{D}{bc^{2}}\left(\lambda \left(\varepsilon -2\left(1+\alpha -K\left(\psi \left(x\right),I_{t}\right)\right)\right)+\beta \left(1-\mu \left(x\right)\right)\right)$$

## 4. Design Flow Description

In order to achieve useful and competitiveness models, tools and methodologies are the fundamental elements of any design flow for industrial or academic embedded systems. These methodologies and tools are usually properly joined up according to a design space exploration framework in order to facilitate the discovery, the management and the evaluation of the design alternatives prior to being implemented. In our case, the target algorithm is hard to specify by using conventional hardware description languages, *i.e.*, Verilog or VHDL. Handling the code of such an application would be far from easy due to the fact that register-level languages requires a high code density for low-level hardware configuration or behavior specification. Therefore, finding an effective design flow will help for reducing the complexity of the design and for increasing its maintainability, as well.

Recently, numerous research works pointed out that high-level synthesis is an effective method to reduce the complexity of FPGA designs. For example, Wakabayashi Kazutoshi [30] shows that a 1 M-gate (Gate count refers to the quantity of the *NAND* equivalent gate in ASIC. This value is usually used to estimate the complexity of the design.) design usually requires about 300 K lines register-transfer level code, while the code density can be easily reduced by 7−10× when moved to high-level specification in C-like languages, resulting in a much reduced design complexity. Villarreal *et al.* [31] present a Riverside Optimizing Compiler for Configurable Circuits (ROCCC) that achieves an average improvement of 15% in terms of the metrics of lines of code and programming time over hand-written VHDL in evaluation experiments. Consequently, we first propose a design flow and its tool kits, which incorporate the high-level synthesis technique for its advantages of high development productivity. The proposed approach has the following properties:

(a) to allow the exploration in different development environments and hardware.
(b) to handle the algorithm analysis and top level simulation in a very high level language, MATLAB.
(c) to handle the algorithm prototype written in C without FPGA expertise or even not for FPGAs, but the platforms of other types.
(d) to optimize the performances of the designs with the hardware constrains, such as frequency or area of the target device.
(e) to automatically generate the desired RTL implementation in a short time, rather than hours or even days.
(f) to be capable of being implemented by using the currently-available electronic design automation tools. This is important for industrial designs, because it can effectively reduce the R&D cost by helping with fast building the desired development platforms and avoiding the additional cost for the new tool kits.

**Figure 2:**
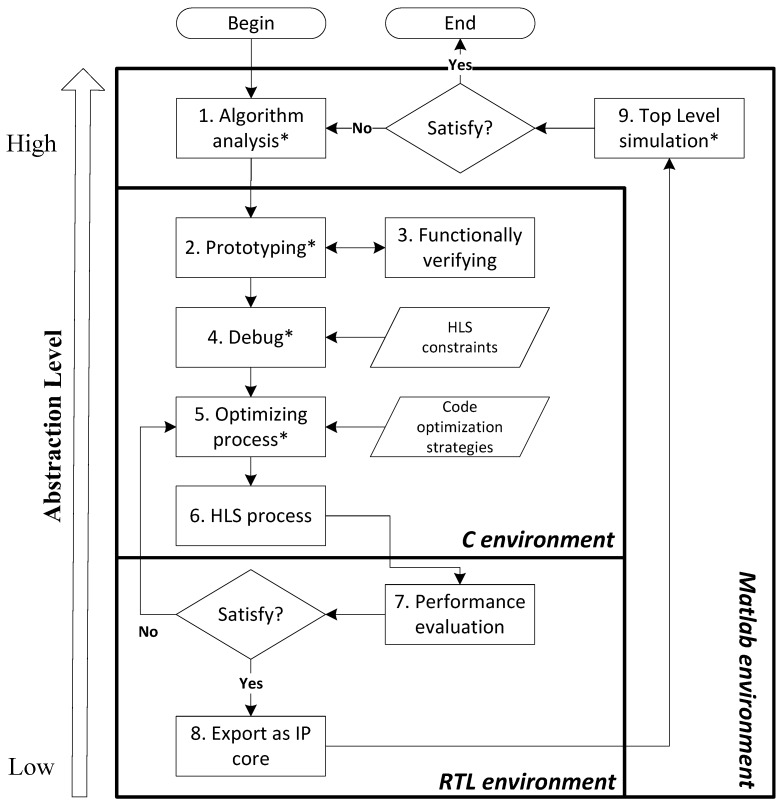
High-Level Synthesis (HLS)-based design flow: “ * ” refers to the manual-necessary cycles.

Figure 2 shows the proposed design flow. Considering most designs begin with the algorithm analysis, which requires a very high level environment to facilitate the descriptions of mathematical operations and the simulations of the model, we select MATLAB as the favorite user-level design environment for its advantages in the terms of vector processing and powerful built-in image processing tools. Once the algorithm is verified, we can start to prototype the algorithm for FPGA synthesis. Thanks to the HLS technique, the algorithm behavior can be prototyped in the C environment and verified by using the common C compilers. It should be noted that the original prototype has to be debugged depending on the input code constraints of high-level synthesis due to the special architecture of FPGAs compared to general-purposed processors. Before running the synthesis, some optimizations can be made to improve the performances of the design (the optimization strategies applied in this paper will be discussed later). The C-to-RTL conversion of the development is automated via a high-level synthesis process. Finally, the generated code is evaluated by using a hardware description language estimator to determine whether it satisfies the performance requirements. If satisfied, the generated implementation is then exported as an IP core to the test bench for top level simulation; otherwise, the process of code optimization is iterated again.

We implement the development platform by using five different design tools within Ubuntu, including MATLAB, Gedit, ICC, AutoELS and System Generator (see Table 1), which can benefit both the development productivity and design performance according to their different development environment. In our work, MATLAB and System Generator are respectively used for algorithm analysis and top level simulation of the generated kernel for their advantages of high-level abstraction. Meanwhile, Gedit is selected as the code editor for prototyping and optimizing in a C environment, while ICC for simulating the source code by using general-purposed processors. The gap between C and register-level languages is bridged via AutoELS, which is one of the leading high-level synthesis tools for its abilities to produce high quality register-level languages [32].

Within this design flow, all of the manual, necessary cycles, including algorithm analysis, prototyping, debug, optimizing and top level simulation, are made in a high-abstraction environment, while the C-to-RTL conversion is automated by incorporating the HLS process. This is an effective approach to free the users from the boring work of hardware configuration required at the low abstraction level. Moreover, the proposed approach allows a design space exploration in different environments (MATLAB, C and System Generator) and processors (CPU, FPGA and GPU), which provides a large number of opportunities to find the potential design solutions compared to the design flows targeted to only a single type of environment or device.

## 5. Implementation and Optimization

This section describes the implementation and optimization issues of the proposed very high resolution image segmentation design. Algorithm 1 shows the pseudocode of the original version of the target algorithm in Kintex7_fpv6 from Xilinx. This implementation is first made and functionally verified depending on the algorithm analysis results presented in Section 3 without any hardware level optimizations, then debugged according to the HLS source code constraints for the C-to-RTL conversion.

**Algorithm 1** Pseudocode of the original implementation.
**Require:** original image *I*, initial zero level contour *ϕ*
**Ensure:** the final zero level contour *ϕ* 
 1: Initialize the coefficients
 2: **for all** pixels **do** 
 3:      Compute Gaussian memberships with Equation (11)
 4: **end** **for**
 5: Initialize the local textural information
 6: **for all** pixels **do**
 7:      Compute the body force with Equation (23)
 8: **end** **for**
 9:*τ* ← (9.0 × *γ* + 2.0)/4, with *γ* = 1
10: **for all** pixels **do**
11:      Initialize the LBM distribution function
12: **end** **for**
13: **for all** iterations **do**
14:      **for all** pixels **do**
15:           Perform streaming-collisions within the D2Q5 LBM Lattice structure
16:      **end** **for**
17:      **for all** pixels **do**
18:           Update the distance value
19:      **end** **for**
20: **end** **for**


Despite the benefits of high quality designs for small kernels, the performances of the HLS-generated RTLs are still far away from the manual designs for complex applications, even by using the most advanced tool, such as AutoELS. For example, in the cases of Rupnow *et al.* [33] and Liang *et al.* [34], the performance difference between the two is up to 40× for a high-definition stereo matching implementation. Consequently, various academics proposed different solutions to this issue [35]. Cong *et al.* [36] point out that the quality of generated register-transfer languages of HLS is influenced by the high-level description of the language. Meanwhile, Huang *et al.* [37] deeply study the effects of different compiler optimizations on HLS-generated hardware. They demonstrate that the following two factors can significantly improve the quality of generated RTL: the optimizing itself and its applied ordering.

In this paper, the original version of the proposed implementation shown in Algorithm 1 results in an inefficient block hierarchy and a complex control behavior, which seriously constrains the potential optimization gains of the design in terms of instruction parallelization. According to our analysis, the primary bottleneck of the optimization of our algorithm is that the loops in the routine cannot be completely unrolled due to the hardware constraints. That results in an inefficient parallelization because the instruction-level optimizations are disrupted by the loop and function hierarchy within HLS process. For example, the loops of Lines 2, 6 and 10 cannot be parallelized, even if they are independent, because the generated register-transfer level implementation is based on the Finite State Machine (FSM) architecture, and the loop bodies are processed as separate states that have to be run one by one.

According to the findings presented above, we innovatively take into account the effects of applying ordering in order to improve the performance of our design. We sequentially apply four different source code optimization strategies onto the target implementation, including Function Inline (FI), Loop Manipulation (LM), Symbol Expression Manipulation (SEM) and Loop Unwinding (LU).

### 5.1. Function Inline

According to the design flow proposed in Section 4, the C prototype of the design needs to be debugged for HLS. Since FPGAs structurally differ from general-purposed processors, neither the static definition, nor the dynamic loop boundary are supported in HLS-available C programming. Thus, one of the primary works of the debugging process is to correctly manage both the static variables and the dynamic loop boundaries in the original code. These constraints even result in that some commonly-used C library functions, *i.e*., *exp()*, *rand()* or *pow()*, are not compliant with HLS. To resolve this issue, we pragmatically build a novel C library that includes a series of alternatives for each of the C functions that are not compatible with HLS. In this work, the *exp_hls()* and pow*_hls() functions are used to respectively substitute the standard *exp()* and *pow()* functions, in which “ * ” indicates “1, 2, 3 ⋯ 10”.

**Figure 3:**
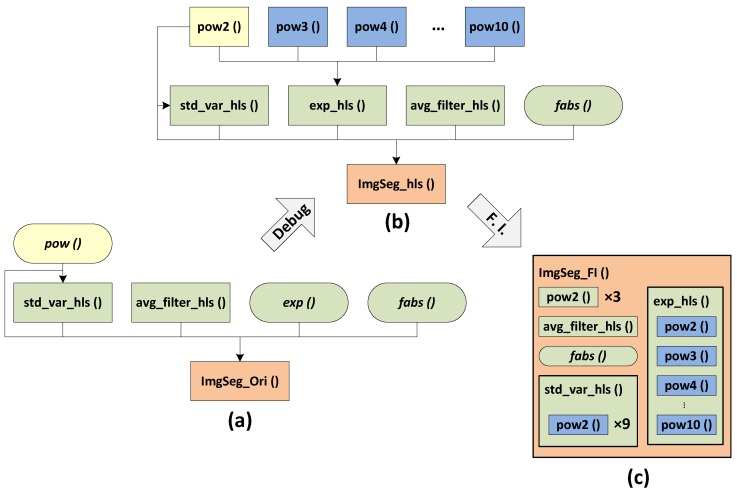
Block hierarchy comparison: (**a**) original version; (**b**) debugged version; and (**c**) Function Inline (FI) version.

However, the approach presented above seriously raises the complexity of the generated RTL. In HLS, the C sub-functions are processed as reusable sub-blocks. Consequently, the sub-functions are separately processed first and then interconnected via assignment interfaces in the functions in the upper level. In this paper, we use the number of assignment to estimate the complexity of the implementations. Figure 3a,b displays the block hierarchies before and after debugging. In this figure, an assignment is represented by a directed wire (e.g., from *pow()* to *ImgSeg_Ori()*), so we can find that the original version leads to only six assignments and increases to fifteen for the debugged version. Despite an optimization possibility by using function-level parallelism, due to the isolation of each sub-block, the optimizations available in the deeper loop and instruction levels are confined to the scope of the blocks even if some different block loops or operations are able to be more efficiently manipulated. Therefore, the first step of the proposed optimization process is to compress the hierarchical architecture of the generated RTL into a single level by substituting the corresponding source code for the sub-function calls in the top function, which is known as function inline. This processing can offer more optimization opportunities by merging the separate loops and operations into a single scheduling scope. Furthermore, for that reason, it is firstly made during the optimization process.

### 5.2. Loop Manipulation

In HLS, the control logic of the source code is first analyzed and extracted as an FSM. Next, the operations are assigned to the corresponding states of the control behavior to perform the control-and-datapath behavior. During this process, either the sequences of operations or the loop bodies are represented as a single state, *i.e*., Lines 5 or 9 for the operation sequence and Lines 3, 7 or 11 for the loop body in Algorithm 1, and the operation scheduling is confined by the state scope.

**Figure 4:**
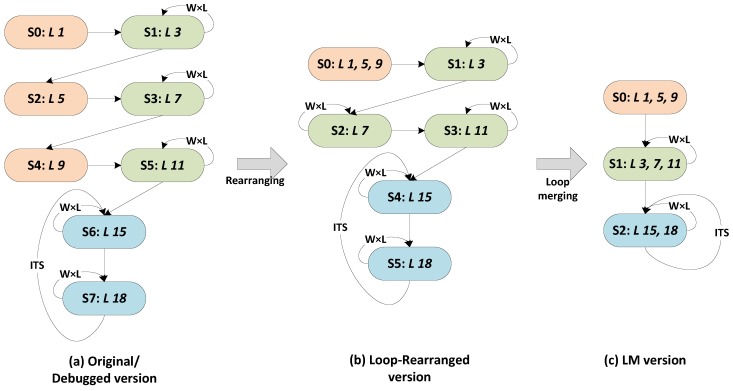
Finite State Machine (FSM) transition within Loop Manipulation: “ L * ” refers to the operations covered in the present state, in which “ * ” indicates the line number in Algorithm 1, W×L=948×450 is the image dimension and ITS is the maximum iteration number defined as five in this paper.

In this paper, applying loop manipulation onto the proposed designs is motivated by two reasons: (1) reducing the running-cost due to the state transit controls by reducing the state numbers; and (2) helping HLS to discovering more instruction-level parallelism opportunities by expanding the scheduling scopes that are confined by states. During this process, the control-and-datapath behavior FSM of the design is optimized by subsequently using operation sequence rearranging and loop merging. The pseudocode of the LM-optimized implementation of this design is shown in Algorithm 2. Figure 4 displays the transition of the generated RTL within this process. After an analysis of data dependency, the operation sequence is rearranged from the math-convenient ordering into the schedule-convenient ordering, which is known as out-of-order compilation in high performance computing. This transformation merges the different state operations (*L 1, 5* and *9*) into a single state and provides more opportunities to the next step of loop merging by locating the separate loops side by side (*i.e*., *L 3, 7* and *11*). Next, the boundary-similar neighboring loops are merged together.

**Algorithm 2** Pseudocode of the Loop Manipulation (LM)-optimized implementation.
**Require:** original image *I*, initial zero level contour *ϕ*
**Ensure:** the final zero level contour *ϕ*
 1: Initialize the coefficients
 2: Initialize the local textural information
 3:*τ* ← (9.0 × *γ* + 2.0)/4, with *γ* = 1
 4: **for all** pixels **do**
 5:     Compute Gaussian memberships with Equation (11)
 6:     Compute the body force with Equation (23)
 7:     Initialize the LBM distribution function
 8: **end** **for**
 9: **for all** iterations **do**
10:     **for all** pixels **do**
11:          Perform streaming-collisions within the D2Q5 LBM lattice structure
12:          Update the distance value
13:     **end** **for**
14: **end** **for**


According to the comparison between Figure 4a,c, the LM process reduces the state number by 62.5% (eight *vs.* three transits). Meanwhile, the state transit number ratio $R_{LM}$ can be expressed as follows:

(25) $$R_{LM}=\frac{2+(1+ITS)\times W\times L}{(6+ITS)+(3+2ITS)\times W\times L}$$

where W×L=948×450 is the image dimension and ITS is the maximum iteration number defined as five in this paper. Given W×L≫6+ITS>2, we can evaluate $R_{LM}\approx 46\%$, and the transit number of the design is therefore reduced by 54% approximately.

### 5.3. Symbol Expression Manipulation

Thanks to the improvements of scheduling algorithms, current HLS tools are enabled one to optimize the operations scheduling with various time or resource constraints. During this process, the parallelism in the design is exposed first according to the data flow graph, next the scheduling algorithm is used to determine the cycle within which the operations can be scheduled.

In the original version of our design, the target algorithm is mathematically described, which leads to a series of long mathematical expressions, including the computations of Exp(x) and $x^{n}$, local mean intensity value $\bar{I}$ and standard variance *s*, shown as follows:

(26) $$\begin{array}{cc} & Exp=\sum_{n=0}^{\infty }\frac{x^{n}}{n!} \end{array}$$

(27) $$\begin{array}{cc} & x^{n}=\prod_{n}x \end{array}$$

(28) $$\begin{array}{cc} & \bar{I}=\frac{1}{N}\sum_{i=1}^{N}I_{i} \end{array}$$

(29) $$\begin{array}{cc} & s^{2}=\frac{1}{N}\sum_{i=1}^{N}{\left(I_{i}-\bar{I}\right)}^{2} \end{array}$$

**Figure 5:**
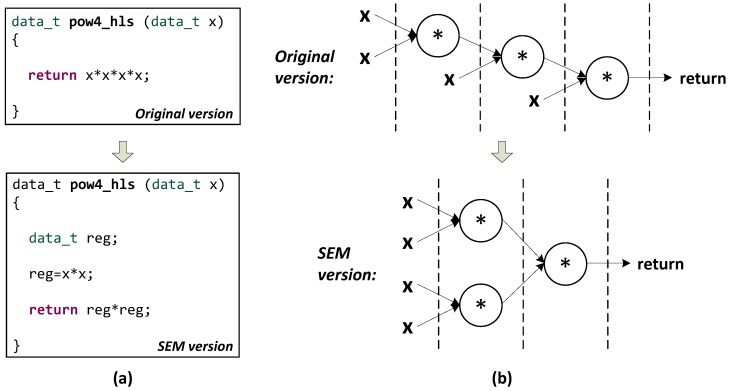
Scheduling of *pow4_hls*: (**a**) original-to-Symbol Expression Manipulation (SEM) code transformation; (**b**) scheduling comparison.

Such mathematical functions seriously prevent designs from benefiting from the instruction-level parallelism optimizations. According to our tests, the parallelism underlying the single equations cannot be effectively exposed by the data flow analysis performed by AutoELS. Therefore, in the symbol expression manipulation, we transform the long expressions in the design into short ones to help HLS to discover more potential parallelizing opportunities. Figure 5 illustrates the code of the function *pow4_hls()* before and after the symbol expression transformation, as well as its scheduling graph. Obviously, the segmented expressions lead to a more efficient implementation. We respectively apply this approach to the descriptions of Equations (26)–(29) and achieve speedups from 24.3% up to 60%.

### 5.4. Loop Unwinding

Loop unwinding is a loop-level parallelism form of optimization widely used in parallel computing. It unwinds first the loops in the source code and next pipelines the body operations for acceleration if their iterations are independent. By using this transformation, engineers can multiply the running speed of the design up to several hundred times. Meanwhile, it should be noted that this method is seriously constrained by the hardware resources of the target device. The area of LLP optimized version $A_{llp}$ can be expressed as follows:

(30) $$A_{llp}=\sum_{k=1}^{K}\left(DOP_{k}\left(n\right)\times a_{k}\right)+A_{control}$$

where *K* is the number of the operation types, $DOP_{k}\left(n\right)$ is the degree of parallelism of the *k*-th operation with the unrolling times of *n*, $a_{k}$ is the area of the *k*-th component and $A_{control}$ is the area of the control circuit. According to Equation (30), the area constraint of loop unwinding can be formulated as:

(31) $$\begin{array}{cc} \; & A_{llp}<A\\ \Rightarrow \; & \sum_{k=1}^{K}\left(DOP_{k}\left(n\right)\times a_{k}\right)<A-A_{control} \end{array}$$

where *A* is the available area of the target device.

Besides pipelining the operations, loop unwinding reduces, as well, access conflicts by enabling the fused iterations to share registers. Since the loop bodies are extracted as independent states in RTL, each of them has to access to the memory individually even if some of the data can be shared by multiple iterations. Fusing separate iterations into a single one breaks the isolation between the loop iterations, which provides a nice opportunity to reduce access conflicts. However, it should be noted that the efficiency improvement from registers sharing among iterations is effective only when the reading operations lead to more delays than the writing operations; otherwise, the accelerations achieved by reading operations reducing will be completely offset by the delays due to writing access conflicts.

Algorithm 3 shows the pseudocode of the LU-optimized implementation of this design. Due to the resource constraints of the target device, we only partly optimize the final nest loop in Line 10 with n=4 by using the *loop unroll* and *pipeline* directives in AutoELS. Our tests demonstrate that this optimization reduces the latency of the target loop by 90.5%.

**Algorithm 3** Pseudocode of the Loop Unwinding (LU)-optimized implementation.**Require:** original image *I*, initial zero level contour *ϕ***Ensure:** the final zero level contour *ϕ*Initialize the coefficientsInitialize the local textural informationτ←(9.0×γ+2.0)/4, with γ=1**for all** pixels **do**    Compute Gaussian memberships with Equation (11)    Compute the body force with Equation (23)    Initialize the LBM distribution function**end** **for****for all** iterations **do**    **for all** pixels **do**        *#pragma AP unroll factor=16*        *#pragma AP pipeline*        Perform streaming-collisions within the D2Q5 LBM lattice structure        Update the distance value    **end** **for****end** **for**

## 6. Experiment

In order to obtain an unbiased conclusion, we compare the proposed implementation with its original RTL implementation and two CPU implementations. These reference implementations are developed from the same pseudocode shown in Algorithm 1. For the sake of fairness, all of the reference implementations are specified within the high-abstraction environments MATLAB or C/C++. Table 2 details their specifications. *cpu_m* is the original implementation after the algorithm analysis; *cpu_c* is its C/C++ version; *fpga_hls_ori* is the RTL generated from the source code without any optimization; and *fpga_hls_opt* is the proposed one of this paper. Since the algorithm is complex, a large FPGA platform, Kintex 7, is selected for evaluation.

In this section, the user-controlled parameters in the algorithm are defined first. Next, the effects of the proposed optimization methods presented in Section 5 are analyzed one by one. Thirdly, the proposed and reference implementations are functionally verified by using four very high resolution satellite images taken by IKONOS or GeoEye-1. Finally, they are evaluated through the running-cost performance comparison.

### 6.1. Parameter Configuration

**Figure 6:**
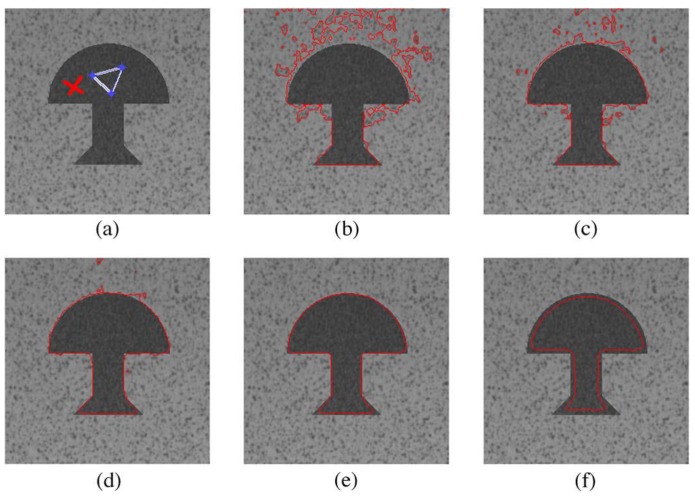
Impact of the parameter *α* on the accuracy of the segmentation result (*cf*. [14]): (**a**) initial contour; (**b**) α=0.5 and β=3.5; (**c**) α=0.75 and β=3.5; (**d**) α=1 and β=3.5; (**e**) α=2 and β=3.5; (**f**) α=10 and β=3.5.

**Figure 7:**
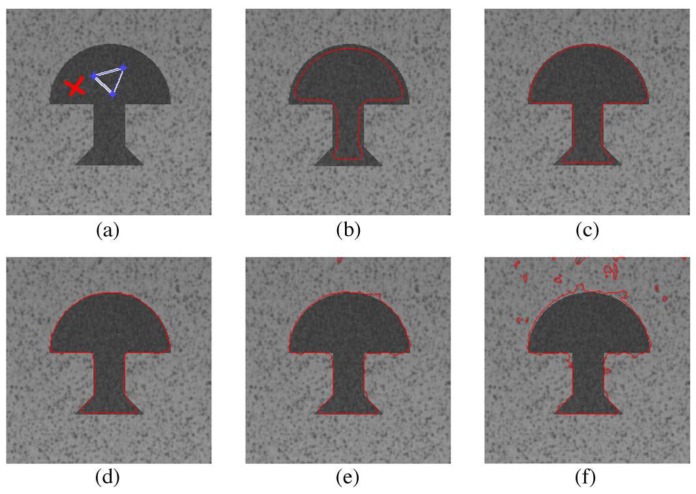
Impact of the parameter *β* on the accuracy of the segmentation result (*cf*. [14]): (**a**) initial contour; (**b**) α=2 and β=1; (**c**) α=2 and β=2.5; (**d**) α=2 and β=3.5; (**e**) α=2 and β=4; (**f**) α=2 and β=5.

In Equation (24), since a D2Q5 model is used in the design, we have D=2 and b=5. The length of each link in the active contour model is defined as 1, so c=1. The parameter *λ* can be used to accelerate the convergence of the proposed method toward the steady state, and users have to manually configure it for different input images. *τ* is the relaxation coefficient that is used to control the curvature, and we fix it to 2.75. *α* and *β* control the impact of the texture information on the segmentation results. In order to segment the text region of interest well, we find the suitable values of *α* according to observing the segmentation results in a test image [14]. In the experiment result analysis of Figure 6, we can see that a too low value of *α* (α=0.5) leads to an over-segmentation, while a too high value (α=10) decreases the precision of the result (under-segmentation). In Figure 7, we can also see that a too low value of *β* leads to an under-segmented result as the curve fails to detect the right contour. Therefore, we fixed α=2 and β=3.5. However, it should be noted that this configuration is obtained within double floating point numbers; for the single floating point and fixed point numbers, we changed *α* from 2 to 5 and 9, respectively.

### 6.2. Optimization Evaluation

Thanks to the proposed design flow, we can easily discover and evaluate different design alternatives. In this paper, we subsequently apply different optimization methods onto the design depending on the nature of the HLS process. Table 3 details the running-cost estimation of each optimization cycle.

In Table 3, it is first seen that each optimization made on the design can effectively reduce the latency of the generated RTL. Their latency improvements related to the previous optimization cycle are respectively 14.5%, 5.1%, 8.7% and 92.7%, while the total is 94.6%. We can find that the main contributor of the overall optimization process is loop unwinding. This is because the LBM solver of this paper has a low iteration dependency, which enables the potentially high parallelism to be exploited. Therefore, the loop unwinding can multiply the running time with few data dependency constraints.

Meanwhile, it is seen as well that the hardware resource consumption varies greatly depending on the different optimization forms. FI and LM accelerate the design, as well as reduce the resource consumption. This is because they can create more operator sharing opportunities by expanding the scheduling scope confined by the function and loop hierarchy, respectively, and reduce the architectural complexity of the design, as well. In contrast, SEM and LU do not have the same capability; therefore, the generated RTLs optimized by them lead to more consumed resources. In this design, the target device is *xc7k70tfbg484-1* of Kintex-7 from Xilinx, and LUTs are the main resource constraints in this design. Due to this constraint, we can only partly unwind the loop as presented in Section 5.4. The LUTs required for each optimized implementations are around 55%, 40%, 38%, 50% and 81%, respectively.

Finally, it should be noted that our design is constrained by the system frequency, because high parallelized implementations may result in long clock periods in FPGAs. The clock column of Table 3 shows the minimum clock periods for each implementation estimated by using AutoELS. We can see that the LU-optimized implementation requires a much longer period than the others. Generally, the minimum clock period of the system, $T_{min}$, can be estimated as follows:

(32) $$T_{min}=T_{co}+T_{delay}+T_{setup}$$

where $T_{co}$ is the delay of the flip-flop, $T_{delay}$ is the delay of the combinatorial logic circuits and $T_{setup}$ is the setup time of the flip-flop. Since $T_{co}$ and $T_{setup}$ are decided by the technology of the target device, $T_{min}$ is only effected by the complexity of the combinatorial logic circuit in our case. As mentioned above, the complexity of the design is reduced from the original implementation to LM, which allows a smaller clock period. However, from SEM, the circuit becomes more and more complex, especially with the loop unwinding. Furthermore, all of the optimizations are made in the same behavior block, which results in a huge and complex single combinatorial logic circuit. Therefore, within SEM and LU, a significant increase of the clock period is produced.

**Figure 8:**
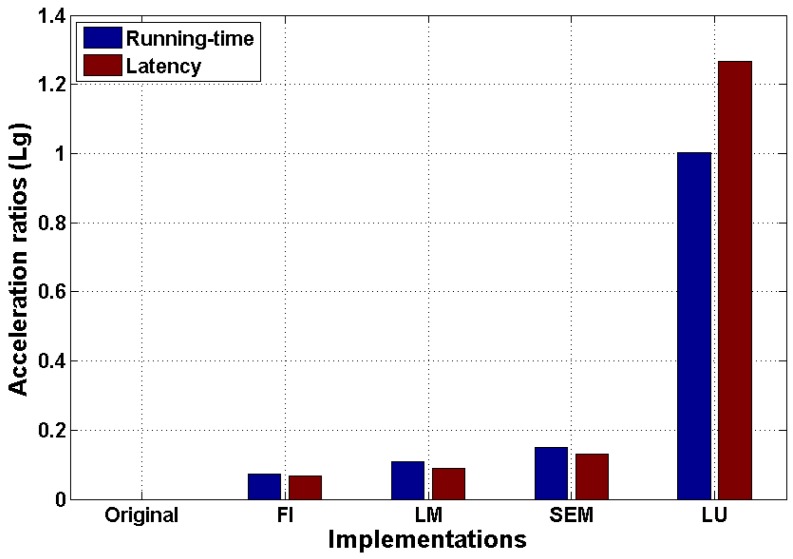
Running time and latency acceleration improvement of different optimized implementations.

Figure 8 compares the running-time acceleration ratio to the one of latency with a logarithmic scale. In this comparison, the original implementation is set as the reference. We can see that, although the efficiency performance of the design is negatively influenced by the frequency constraint, thanks to the significant performance gain of LU, the proposed design achieves a speedup of around 10× compared to its original version.

### 6.3. Function Verification

**Figure 9:**
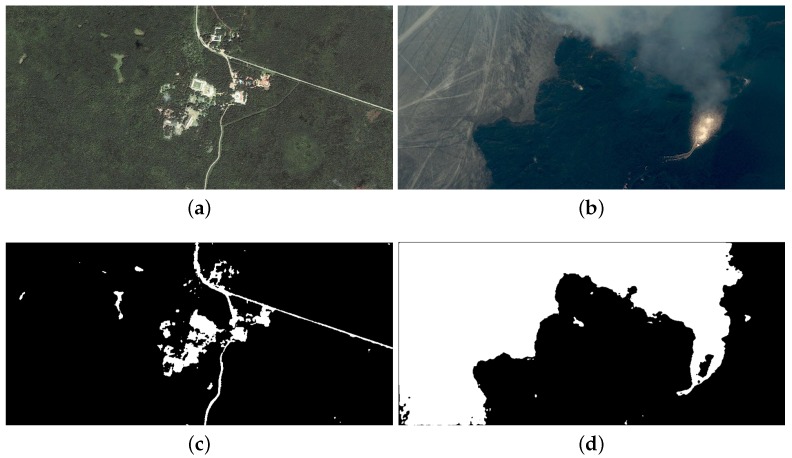

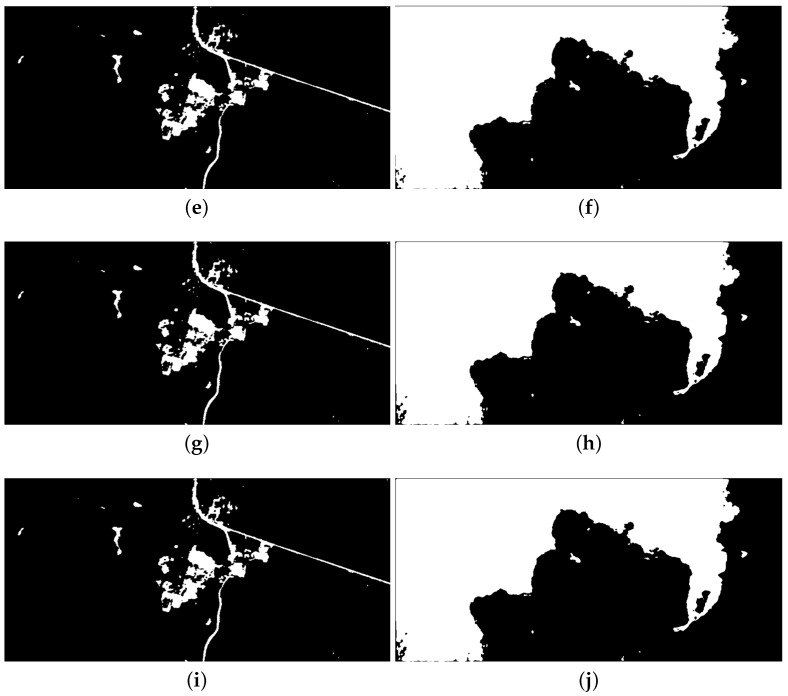
Original images and segmentation results: taken by the IKONOS satellite. (**a**) Original image of Uxmal; (**b**) Original image of volcano; (**c**) *cpu_m* segmentation of Uxmal; (**d**) *cpu_m* segmentation of volcano; (**e**) *cpu_c* segmentation of Uxmal; (**f**) *cpu_c* segmentation of volcano; (**g**) *fpga_hls_ori* segmentation of Uxmal; (**h**) *fpga_hls_ori* segmentation of volcano; (**i**) *fpga_hls_opt* segmentation of Uxmal; (**j**) *fpga_hls_opt* segmentation of volcano.

**Figure 10:**
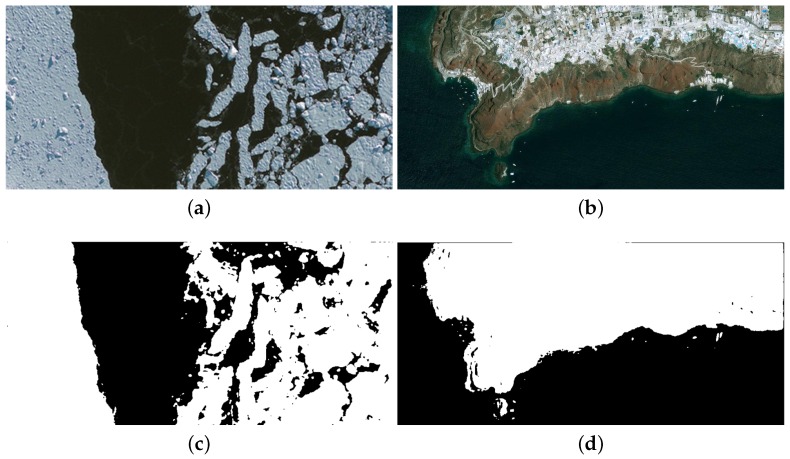

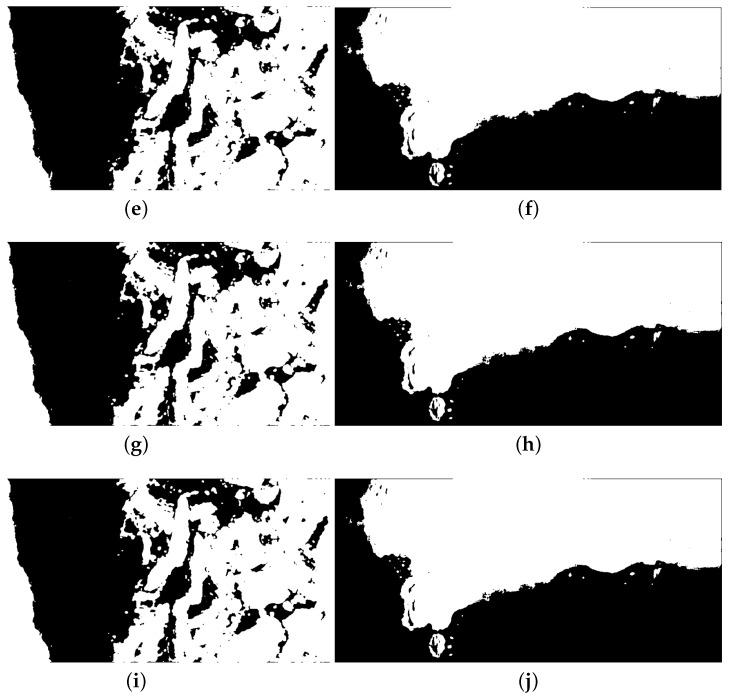
Original images and segmentation results: taken by the GeoEye-1 satellite. (**a**) Original image of ice sheet; (**b**) Original image of Santorin; (**c**) *cpu_m* segmentation of ice sheet; (**d**) *cpu_m* segmentation of Santorin; (**e**) *cpu_c* segmentation of ice sheet; (**f**) *cpu_c* segmentation of Santorin; (**g**) *fpga_hls_ori* segmentation of ice layer; (**h**) *fpga_hls_ori* segmentation of Santorin; (**i**) *fpga_hls_opt* segmentation of ice layer; (**j**) *fpga_hls_opt* segmentation of Santorin.

Figure 9 and Figure 10 show the original images and segmentation results of the reference and proposed implementations. Figure 9a is the image of Uxmal in Mexico taken by the IKONOS satellite in 2002. Its segmentation results demonstrate that the algorithm of this paper can effectively delimit the non-forested areas and extract the road underling the forest. Figure 9b is the image of the volcano in Iceland taken by IKONOS, as well. We can see that the activity of the volcano is well detected. The two photos of Figure 10 were taken by the GeoEye-1 satellite, and the ice-covered areas and the beach line are set as the intended object for the segmentations, respectively. The results demonstrate that the desired areas are well delimited despite the disturbances of the unwanted areas, *i.e*., the convex ices in Figure 10a or the constructions on shore in Figure 10b.

On the other hand, it is seen as well that the segmentation results of the different implementations are highly similar. The similarities between the proposed and reference implementations are quantitatively evaluated by using the *corr2()* function within MATLAB and shown in Table 4. This demonstrates that the selected HLS tools is effective to automate the C-to-RTL transplantation with an acceptable difference, and the optimizations made on the *fpga_hls_ori* would not effect the functions of the design.

### 6.4. Performance Comparison

**Figure 11:**
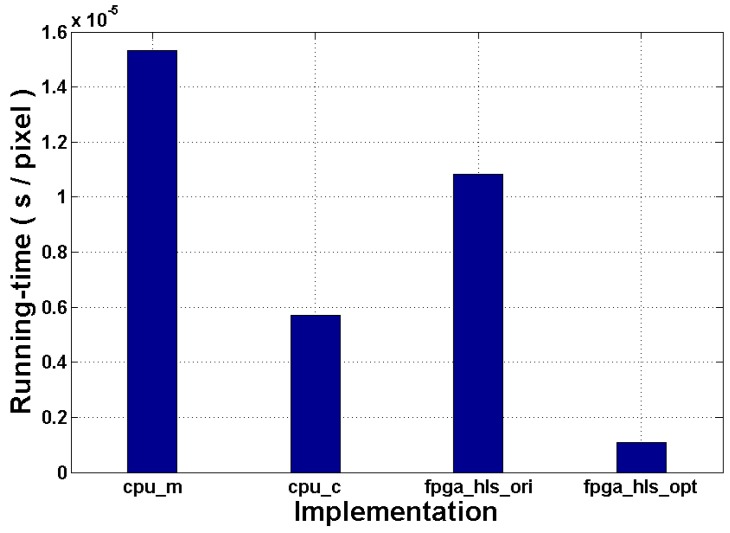
Running time comparison.

This experiment estimates the overall running-time of the proposed and reference implementations in the real world. The scope of this measurement covers the necessary computations and memory access operations. For CPU implementations, specific storage space is allocated in the memory for the input and output images, whereas the external RAM is used for the implementations of FPGA. The results of this experiment are compared in Figure 11.

First of all, we can see that *cpu_c* speeds up the design by around 2.69× related to *cpu_m*. Unlike MATLAB, C/C++ allows design optimization in a lower abstraction level, and a series of compiling tools are developed to automate this process. In this implementation, we compile the source code by using the Intel C++ Compiler. This compiler is able to analyze and vectorize the data flow in order to benefit from Streaming SIMD Extension instructions. It also optimizes the loops by helping OpenMP (Open Multi-Processing) or auto-vectorization to make effective use of caches and memory access, as well [38]. In the compilation process, the “maximize speed” mode is used to schedule the algorithm automatically. This compiler option can create the fastest code in the majority of cases by default. The optimizations made by the compiler include generating intrinsic functions, omitting frame pointer, function inline, *etc*. Therefore, it achieves a significant running time acceleration.

Next, the original RTL of the design *fpga_hls_ori* achieves a speedup of 1.415× related to *cpu_m*. However, it is also observed that *fpga_hls_ori* is 1.9× slower than *cpu_c*. The generated RTL is substantially an FSM coming from the code and data flow graph of the source code, so its operation scheduling has to satisfy the corresponding time constraints. This prevents the design from parallel computing even if some operations are independent and parallelizable. In the experiment of Section 6.2, it is mentioned that this version consumes only 55% of the LUTs of the target device. This demonstrates that it does not make effective use of the additional hardware resource for performance optimization. On the other hand, *cpu_c* is well optimized during the compilation, and the frequency of the target processor Q6600 is around 21× as high as the selected FPGA (2.4 GHz *vs*. 115.2 MHz). All of the reasons mentioned above result in the performance difference of *cpu_c* *vs*. *fpga_hls_ori*.

Finally, we can see that the proposed implementation can achieve the most potential running time gains of all of them. The contributors of this performance gain mainly include the target FPGA device and the optimization method proposed in Section 5. It is known that FPGAs have a high flexibility, which enables users to configure the architecture of the designs as they wish, while the CPU has a changeless architecture, which constrains the operation scheduling, *i.e*., von Neumann bottleneck and the thread number limit. Coupled with HLS, the former can benefit the performance of the design by providing a more efficient architecture and operation scheduling, as well. According to our test, the final RTL optimized by using the proposed method achieves around 14.21×, 5.29× and 10.4× speedups related to the three reference implementations, respectively.

### 6.5. Maintainability

**Figure 12:**
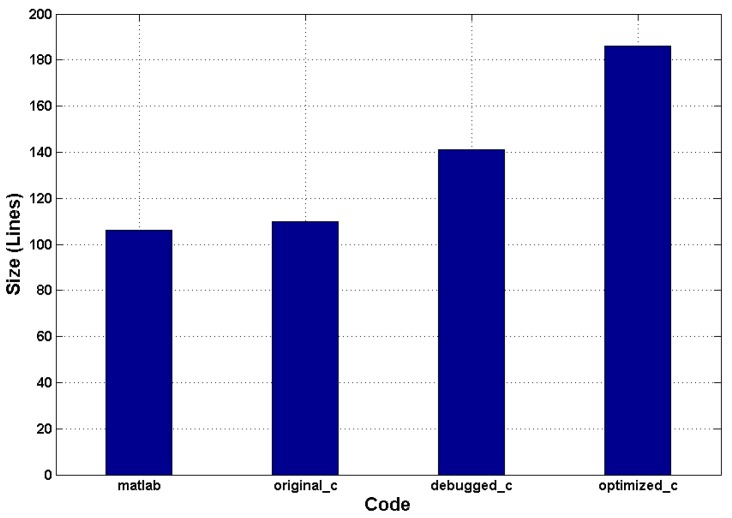
Size estimation of the source code: *matlab* and *original_c* are the MATLAB and C version of the target algorithm, while *debugged_c* and *optimized_c* are the versions debugged and optimized for HLS.

Generally, high-level development environment, *i.e*., MATLAB or C/C++, can add to the maintainability of the design by reducing the size of the source code. The proposed design flow of this work allows a high abstraction environment for FPGA designs. Figure 12 shows the size estimation of the source code for the different implementations. We can see that there is no significant size gap between the *matlab* and *original_c* versions. Meanwhile, *debugged_c* and *optimized_c* necessitate more code for algorithm description. This is because in *debugged_c*, the HLS-unamenable C library functions need to be re-specified manually, while *optimized_c* requires more code to configure the optimization directives or to manage the registers for the proposed optimization methods.

However, it should be noted that this rise is completely acceptable compared to the intensity of RTL code. The code intensity ratio between the hand-written RTL and HLS-generated implementations, $R_{code}$, can be quantified as follows:

(33) $$R_{code}=\frac{L_{RTL}}{L_{HLS}}$$

where $L_{RTL}$ and $L_{HLS}$(=186) refer to the line number of the manual register-transfer level and source C code. $L_{RTL}$ can be expressed by $p\times N_{RTL.gate}$, in which *p* is the ratio between the line number of hand coded RTL and its NAND-equivalent gate count, $N_{RTL.gate}$. According to the findings of Wakabayashi Kazutoshi [30], a 1M-gate design requires 300 K lines of RTL code approximately, which yields p=0.3 lines per gate. The case study of Homsirikamol and Gaj [39] demonstrates that the area ratio between the HLS-generated and hand-written register-transfer level code, *q*, varies from 0.78–1.2 times within the devices of Xilinx, so we compute $N_{RTL.gate}$ by using $q\times N_{HLS.gate}$, in which $N_{HLS.gate}$ is the gate count of the HLS-based implementation. The design of this paper consumes around 1.34 M gates, so the proposed implementation may result in 313.56–482.4 K lines of RTL code. We can see that the proposed research and development method can effectively improve the maintainability of the design by greatly reducing its dimension by around 1686–2594 times related to the conventional method.

## 7. Conclusions

In this paper, we implement an embedded VHR satellite image segmentation design. First of all, we select FPGAs as the target device for the desired design and built a novel dedicated design framework by using the HLS technique. The proposed design flow can effectively accelerate the development cycles by facilitating the algorithm analysis, the design implementation and the exploration of design alternatives. Next, an active contour model and its LBM-based solver are prototyped and analyzed as the target image segmentation algorithm in a math-convenient environment. The proposed algorithm has a high parallelism nature, which can greatly improve the design performance. During the implementing process, the design is optimized depending on the features of HLS.

In the evaluation experiments, it is seen that the proposed design can produce a high quality image segmentation result in the nature or disaster images taken by IKONOS or GeoEye-1. Compared to the reference implementations on the CPU or FPGA, the proposed design has a 5.29–14.21× higher running time performance with a similar capacity in terms of maintainability. Therefore, it is concluded that the achievement of this paper has a great application prospect and potential in the remote sensing-based nature disaster prevention and monitoring.

In this work, we find as well that the performance of the generated RTL is seriously constrained by the hardware areas. Meanwhile, the GPU may provide a higher parallelism degree because it is capable of running hundreds of threads simultaneously. However, the platform of this type is always challenged by the bottleneck of the host-device communication cost and device memory capacity. For this issue, the CUDA-aware Message Passing Interface framework will be considered to further improve the desired application. To do this, the algorithm will be re-analyzed to perform a highly efficient task scheduling. Additionally, the HLS-based design flow results in a high intensity Verilog code for the design of this paper, and our experiments demonstrate that the changes of data formats may produce small and acceptable impacts on the image segmentation results. Therefore, analyzing the basic architecture of the design in the register-transfer level will help to understand how it works and may give considerable improvements both in terms of clock speed and logic resources required.

## Figures and Tables

**Table 1:** Tool kits of the proposed design flow within Ubuntu.

Number	Cycle Name	Tools
1	Algorithm analysis	MATLAB
2	Prototyping	Gedit
3	Functionally verifying	ICC
4–5	Debug, Optimizing process	Gedit
6–8	HLS process, performance evaluation, IP-core exporting	AutoESL
9	Top level simulation	System Generator

**Table 2:** Implementation specifications: “ * ” indicates the proposed implementation.

Implementations	Environments	Data Formats	Tools	Target Devices
cpu_m	MATLAB	64-bit double floating point	MATLAB R2012a	Intel Q6600
cpu_c	C/C++	32-bit floating point	ICC 13.1.1	Intel Q6600
fpga_hls_ori	C/C++	32-bit fixed point	AutoELS	Xilinx Kintex 7
fpga_hls_opt *	C/C++	32-bit fixed point	AutoELS	Xilinx Kintex 7

**Table 3:** Optimization evaluation: “ * ” indicates the proposed implementation. LM, Loop Manipulation; LU, Loop Unwinding.

Optimization Cycles	Latency (Cycles)	Clock (ns)	BRAMs	DSPs	FFs	LUTs
Original	532,072,992	8.68	3	74	13,462	23,403
FI	454,940,736	8.58	3	43	10,607	16,916
LM	431,875,392	8.33	3	41	9858	16,161
SEM	394,141,984	8.33	3	61	12,287	21,263
LU *	28,772,758	16	0	66	21,422	34,457

**Table 4:** Segmentation result similarities of the proposed and reference implementations: Evaluated by the *corr2()* function within MATLAB.

	Uxmal	Volcano	Ice Sheet	Santorin
cpu_m	0.9025	0.9838	0.9357	0.9736
cpu_c	0.9362	0.9896	0.9949	0.9745
fpga_hls_ori	1	1	1	1
